# Inclusion body myositis and associated diseases: an argument for shared immune pathologies

**DOI:** 10.1186/s40478-022-01389-6

**Published:** 2022-06-03

**Authors:** Christopher Nelke, Felix Kleefeld, Corinna Preusse, Tobias Ruck, Werner Stenzel

**Affiliations:** 1 Department of Neurology, Medical Faculty, Heinrich Heine University Düsseldorf, 40225 Düsseldorf, Germany; 2 Department of Neurology, Charité-Universitätsmedizin Berlin, Berlin, Germany; 3 Department of Neuropathology, Charité-Universitätsmedizin Berlin, corporate member of Freie Universität Berlin and Humboldt-Universität zu Berlin, Berlin, Germany; 4 Department of Neurology With Institute for Translational Neurology, University Hospital Münster, 48149 Münster, Germany

**Keywords:** Inclusion body myositis, Pathophysiology, CD8+ T cells, Immune senescence, Cellular senescence

## Abstract

Inclusion body myositis (IBM) is the most prevalent idiopathic inflammatory myopathy (IIM) affecting older adults. The pathogenic hallmark of IBM is chronic inflammation of skeletal muscle. At present, we do not classify IBM into different sub-entities, with the exception perhaps being the presence or absence of the anti-cN-1A-antibody. In contrast to other IIM, IBM is characterized by a chronic and progressive disease course. Here, we discuss the pathophysiological framework of IBM and highlight the seemingly prototypical situations where IBM occurs in the context of other diseases. In this context, understanding common immune pathways might provide insight into the pathogenesis of IBM. Indeed, IBM is associated with a distinct set of conditions, such as human immunodeficiency virus (HIV) or hepatitis C—two conditions associated with premature immune cell exhaustion. Further, the pathomorphology of IBM is reminiscent of other muscle diseases, notably HIV-associated myositis or granulomatous myositis. Distinct immune pathways are likely to drive these commonalities and senescence of the CD8^+^ T cell compartment is discussed as a possible mechanism of pathogenesis. Future effort directed at understanding the co-occurrence of IBM and associated diseases could prove valuable to better understand the enigmatic IBM pathophysiology.

## Inclusion body myositis—current concepts

At present, IBM is classified among the idiopathic inflammatory myopathies (IIM), which includes dermatomyositis (DM), immune-mediated necrotizing myopathy (IMNM), myositis in antisynthetase syndrome, and a group of non-specific IIM [51]. However, these entities are unlikely to comprise all types of IIM that occur. Among IIM, IBM is unique as it does not occur in children, has a relatively ‘pure’ muscle phenotype, and shows only subtle therapeutic response to contemporary treatments, if at all, making IBM difficult to contextualize immunologically [25]. The presumed presence of degenerative features, such as rimmed vacuoles and protein aggregations, has provoked a longstanding debate regarding the pathophysiology of IBM. The early description of cytotoxic CD8^+^ T cell infiltrates in the endomysium by the late Kichii Arahata were consolidated by further studies of the clonal expansion of CD8^+^ T cells and their T cell receptor (TCR) repertoire in IBM [2, 25]. This line of argumentation has very recently been fostered by the identification of effector memory T cells re-expressing CD45RA (TEMRA) and CD8^+^ T cells with an exhausted phenotype as evidenced by expression of CD57 and KLRG1, among others [26, 27].

Conversely, in-depth analysis of rimmed vacuoles and their content identified a number of proteins, none of which are exclusive to IBM [29]. Nonetheless, there is uniform agreement that the presence of rimmed vacuoles, as they occur in IBM, and their specific morphological features inform about defective macroautophagic pathways [6, 8, 52]. Recent advances in whole genome sequencing covered mitochondrial DNA (mtDNA) to a mean depth of 46,000 × in skeletal muscle specimens obtained from 21 IBM patients [31]. Here, mtDNA deletions and duplication were identified both in IBM and aged controls but were more pronounced in IBM. Indeed, the level of heteroplasmy in IBM was 10% (range 1% to 35%) compared to 1% in controls (range 0.2% to 3%). The similarity to patterns observed in mtDNA polymerase gamma A catalytic subunit (Pol□A)-associated (*POLG-associated)* disease allows the hypothesis that there is a defective mtDNA replication machinery in IBM muscle resulting in accelerated aging driven by chronic inflammation. Mitophagy is a specific autophagy program eliminating dysfunctional mitochondria, thereby contributing to cellular homeostasis [13]. However, altered protein levels of receptors necessary for effective mitophagy were previously described in IBM [59]. Indeed, Nogalska et al. observed that the function and expression of Bnip3, a key receptor for effecting mitophagy, is preserved—and perhaps increased—in sporadic IBM. The authors suggest that impaired lysosomal function and mitochondrial enlargement contribute to ineffective mitophagy, contributing to the accumulation of damaged mitochondria seen in IBM [31, 59].

**Fig. 1:**
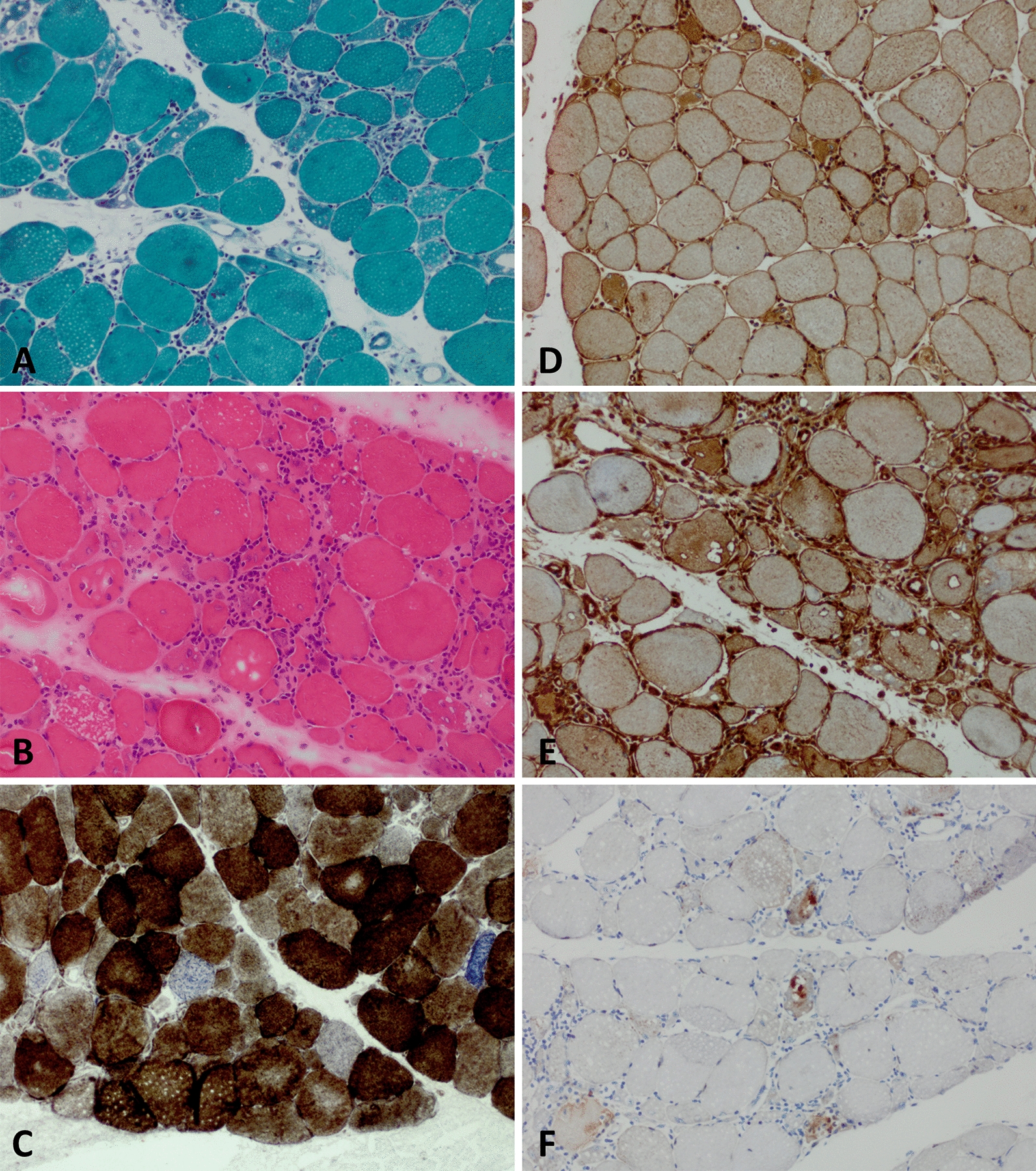
Characteristic pathomorphology of IBM. Pathomorphological characteristics of IBM patients as seen on muscle biopsy. (**a**) Pronounced fiber size variation with hypotrophic and hypertrophic fibers as well as internalized nuclei, myofiber necrosis and endomysial lymphocytic infiltrates and rimmed vacuoles. Gömöri trichrome staining (× 200). (**b**) Pronounced fiber size variation with hypotrophic and hypertrophic fibers as well as internalized nuclei, myofiber necrosis, endomysial lymphocytic infiltrates and rimmed vacuoles. H&E staining (× 200). (**c**) Presence of COX-negative, SDH-positive myofibers. COX-SDH staining (× 200). (**d**) Myofibers display sarcolemmal (and sarcoplasmic) positivity for MCH class I. MHC class I staining (× 100). (**e**) Myofibers display sarcolemmal (and sarcoplasmic) positivity for MHC class II. MHC class II staining (× 100). (**f**) Coarse p62^+^ autophagic material mostly localized in vacuoles. p62 staining (× 200). *COX*  cytochrome oxidase immunohistochemistry; *H&E*  hematoxylin and eosin; *IBM*  inclusion body myositis; *MHC*  major histocompatibility complex; *SDH*  succinate dehydrogenase

The prototypical pathomorphology of IBM comprises four major categories that were first explored in 1978 by a brilliant description from the late Stirling Carpenter [12] and were now complemented by modern molecular analysis (Fig. 1):

1. Highly specific inflammatory features consisting of endomysial T cell infiltrates showing a predominance of CD8^+^ lymphocytes. These lymphocytes exhibit a characteristic pattern of terminal differentiation being positive for the markers KLRG1 and CD57 and losing CD28 expression [27, 40]. They are accompanied by highly differentiated Siglec 1^+^ macrophages co-staining with STAT6 or STAT1 in active myophagocytosis. Further, interferon-signature proteins such as IRF8 and ISG15 are co-expressed on major histocompatibility complex (MHC) class II-positive macrophages in the endomysium as evidenced by proteomic and immunohistochemical analysis [64]. In this context, the sarcolemma of most myofibers are MHC class I and II positive, while complement depositions are likely unspecific [4].
2. Rimmed vacuoles and a range of misfolded proteins either associated with the vacuoles or lying beneath the myofibrils. Vacuoles may be scarce, but more often, they are identified on consecutive levels of the muscle specimens. They are most easily identified with p62 or LC3 [8]. The pentameric form of formyl thiophene acetic acid (pFTAA) stains as coarse plaque-like deposits and highlights defective (macro)-autophagy [39, 52]. Of note, amyloidogenic deposits (misfolded proteins with a β-pleated structure) must not be mistaken for amyloid-β, which is processed by secretases and shed to the extracellular (not intracellular) space.
3. Mitochondrial damage with ragged-red, -blue or -brown fibers as well as cytochrome c oxidase (COX)-negative (and SDH-positive) fibers. Further, unambiguous ultrastructural signs of abnormal mitochondrial fine structure (e.g. paracristalline inclusions or circular cristae) constitute a hallmark of IBM but can present variably in quality and quantity [31, 46]. The absence of mitochondrial damage renders the diagnosis of IBM highly unlikely.
4. The extent of tissue damage increases over time as characterized by increased fibrous and fatty tissue in the endomysium. Together with marked variability of fiber size and the presence of necrotic fibers, a plethora of structural sarcoplasmic abnormalities such as targetoid defects and coarse sarcolemma appearance on NADH-tetrazolium reductase stains gives a severe ‘myopathic-dystrophic’ appearance [8]. This pattern occurs variably across the course of disease [70].

In short, a distinct histological pattern defines IBM, with mitochondrial dysfunction and muscle infiltrates of an expanded, cytotoxic CD8^+^ T cell population as prominent features.

## Which types of immune association may characterize IBM?

The pathogenesis of IBM has given rise to much speculation and, despite recent advances, remains largely enigmatic. Thus, the immunopathological framework of IBM likely differs from other IIM presenting with an acute immune response. Still, understanding how IBM associates with other diseases might provide insight into shared immune mechanisms, potentially providing a new understanding of the pathogenesis of this unique disorder.

To discuss IBM and its association with other disorders, it must first be clarified how pathologies might co-occur with IIM. First, some IIM exhibit organ involvement other than skeletal muscle as a defining feature. Most notably, these include DM and antisynthetase syndrome myositis. Here, extramuscular involvement is a clinicopathological feature characteristic of the underlying disease. A second group of poorly defined myositis commonly includes patients with overlap forms of IIM, in which muscle inflammation co-occurs with disorders from the spectrum of rheumatological disease [4, 5]. While this group is often summarized as ‘overlap myositis’ (OM), a definition met by international consensus is lacking at presence. Lastly, typically muscle-restricted IIM, such as IBM, might develop in association with other diseases. These disease phenotypes often resemble their idiopathic form both clinically and histopathologically. IBM and IMNM are notable examples for IIM with a predominant and most often ‘pure’ muscle phenotype.

## Polymyositis—an IIM entity at the crossroads

The discussion of IBM and associated pathologies is complicated by the IIM subgroup of polymyositis (PM). In contemporary approaches to classification of IIM, PM remains a poorly defined entity lacking distinct clinical and seropathological diagnostic criteria [50, 51]. A recent retrospective analysis applied the current diagnostic criteria to a cohort of 37 patients previously diagnosed with PM. The diagnosis could be maintained in 9 patients (24.3%), while others were classified as other IIM entities based on serological and histopathological data. These 9 PM patients accounted for 3.5% of the total cohort of 255 IIM patients included in the analysis, indicating that PM might constitute a separate, but rare, subgroup of IIM [48]. Currently, PM remains a point of discussion with some authors arguing for a strict clinicopathological definition, while others advocate for a broader interpretation of PM, allowing for the inclusion of otherwise unclassifiable cases [45]. Some authors also argue for PM belonging to the clinicopathological spectrum of IBM. This notion is exemplified by the concept of PM with mitochondrial pathology (PM-Mito) [4, 60]. The extent of rimmed vacuoles might vary among IBM specimens, with some authors defining patients, that might otherwise be classified as IBM, due to the absence of rimmed vacuoles as having PM-Mito [60, 77]. The available studies do not currently allow for a conclusive statement as to whether PM, PM-Mito and IBM are clearly distinct disease entities or whether they belong to a common spectrum of IIM.

## IBM and the human immunodeficiency virus

The skeletal muscles can be subject to damage during all stages of infection with human immunodeficiency virus (HIV). Broadly, damage to skeletal muscle in association with HIV can be attributed to inflammatory mechanisms or toxicity of anti-retroviral therapy [42]. With respect to the former, the virus itself may provoke ephemeral myalgia during seroconversion, while a pro-inflammatory state results from specific alterations of the immune architecture during the disease that may occur even despite clinically effective treatment. Toxic myopathies with the presence of characteristic mitochondrial damage may occur in response to nucleoside-analogue reverse transcriptase inhibitors (NRTI), such as azidothymidine [22, 43], which inhibits mitochondrial DNA polymerase gamma [15]—a regular component of the combined antiretroviral therapy (cART) of the past. Mitochondrial damage has become uncommon as an adverse effect of HIV treatment. Nevertheless, it is interesting to note that mitochondrial damage exemplified by ragged red fibers and COX-negative fibers are a pathogenic hallmark of tissue damage in IBM [15]. In contrast, muscle damage in response to NRTIs is accompanied by subacute, painful myopathy and increased creatine kinase (CK) levels [22]. These clinical features are unusual for IBM. Similarly, NRTI-associated myopathy presents with neither substantial muscle inflammation, positivity of MHC class I or II for myofibers, nor prototypical features of autophagy. Although NRTI-induced myopathy shares mitochondrial damage as a feature with IBM, it appears that the immune pathology of IBM is more complex, resulting in a distinct clinical phenotype.

**Fig. 2:**
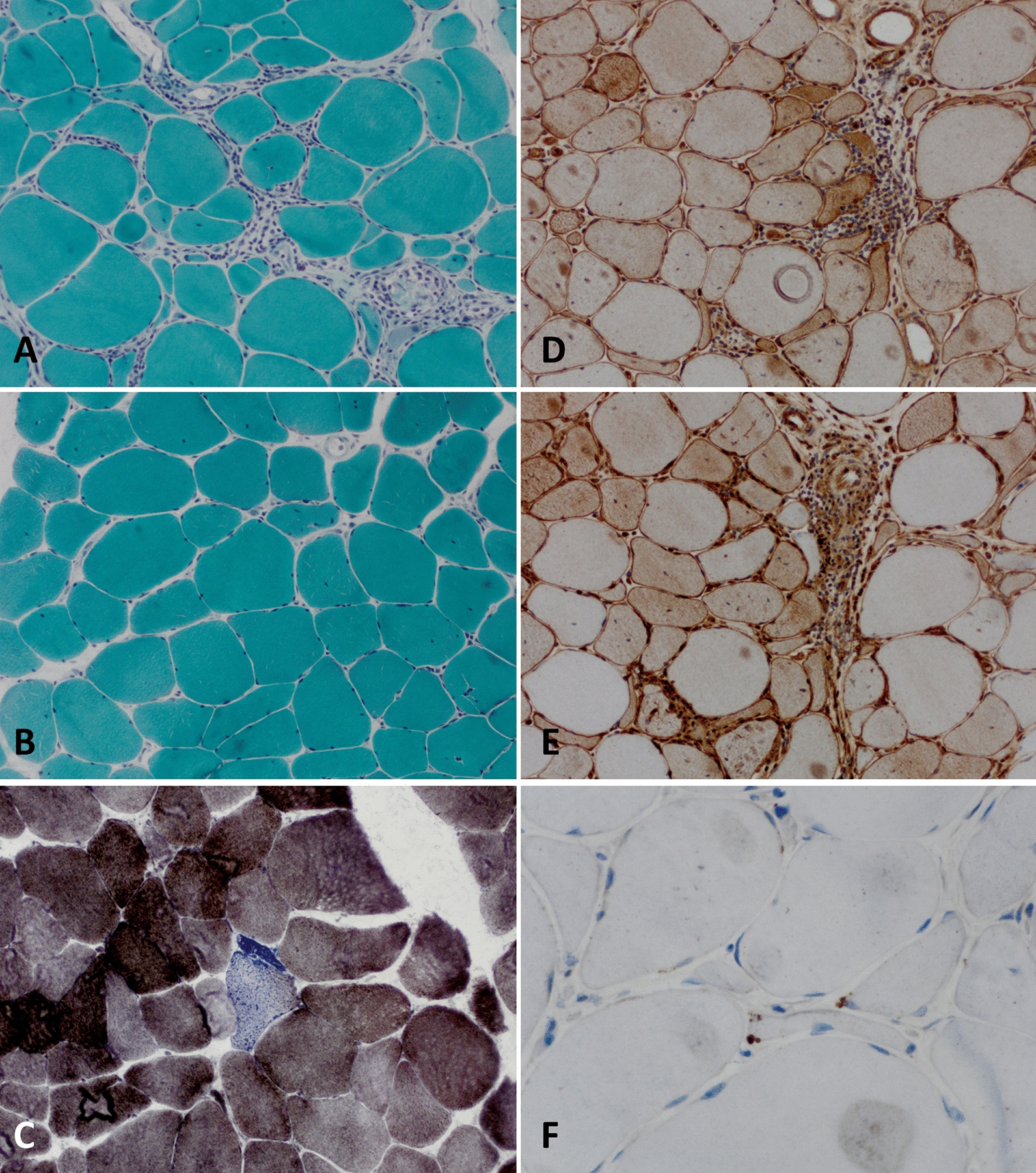
HIV-associated IBM. Pathomorphological characteristics of IBM associated with HIV as seen on muscle biopsy. (**a**) Myopathic picture with pronounced fiber size variation with hypotrophic and hypertrophic fibers and endomysial lymphocytic infiltrates. No overt rimmed vacuoles are seen. Gömöri trichrome staining (× 200). (**b**) In other ares of the same biopsy specimen, a milder myopathic picture is evident with only single lymphomonocytic cells in the endomysium. No overt rimmed vacuoles are seen. Gömöri trichrome staining (× 200). (**c**) Presence of COX-negative and SDH-positive myofibers. COX-SDH staining (× 200). (**d**) Myofibers display varying sarcolemmal (and sarcoplasmic) positivity for MCH class I. MHC class I staining (× 200). (**e**) Myofibers display varying sarcolemmal (and sarcoplasmic) positivity for MHC class II. MHC class II staining (× 200). (**f**) Single small fibres with initial coarse p62^+^ autophagic material mostly localized subsarcolemmaly and in perinuclear areas (× 600). *COX*  cytochrome oxidase immunohistochemistry; *HIV*  human immunodeficiency virus; *IBM*  inclusion body myositis; *MHC*  major histocompatibility complex; *SDH*  succinate dehydrogenase

In addition to toxicity, HIV-positive patients may develop a distinct inflammatory myopathy reminiscent of IBM. In a retrospective trial, 11 out of 1562 patients with IIM were positive for HIV [47]. It is curious to note that initially, these patients presented with a PM phenotype featuring high CK level and both proximal and distal muscle weakness. Eventually, these patients progressed to an IBM-like phenotype with distinct weakness of the finger flexors, knee extensors and ankle dorsiflexors [47]. Muscle biopsies were characterized by rimmed vacuoles and endomysial inflammation but lacked perifascicular atrophy (Fig. 2). Clinico-pathological progression from HIV-PM to HIV-IBM was emphasized by a consecutive study [33]. The sporadic occurrence of PM-Mito in the context of HIV infection was similarly described to progress to an IBM-like phenotype in a number of studies [70, 77]. Of note, only the age at manifestation was different between HIV-IBM (51 years) and sporadic IBM (69 years), while clinical and histopathological features were reported to be similar [33]. It is tempting to speculate that the change of the clinical phenotype from PM-like to IBM-like is mirrored by chronic stimulation of the adaptive immune system, eventually resulting in an exhausted immune phenotype summarized as immune senescence [58]. The intriguing interplay between IBM and immune senescence will be discussed later in this review.

Early reports on muscle pathology in cohorts of HIV-infected patients described tubuloreticular inclusions in endothelial capillary cells similar to those in DM—perhaps reflecting early effects of interferon signaling on the endoplasmic reticulum—as a hallmark feature of HIV myopathy [43]. ‘HIV myopathy’ has been used as an umbrella term including both toxic (cART-related) and inflammatory myopathies in the context of HIV infection. In a follow up study on HIV myopathy, skeletal muscle biopsies from 46 HIV-positive patients were categorized into five subgroups according to European Neuromuscular Center (ENMC) criteria [42]. Here, IBM accounted for 3 patients, while 18 were classified as PM, 1 as IMNM and 12 as non-specific myositis. In addition, 12 patients displayed isolated mitochondrial abnormalities with COX-negative fibers and without rimmed vacuoles or inflammation [42]. Of note, virus-specific antigens were not detected and only a minor subset of T cells were found to be clonally expanded [16]. In addition to inflammatory features, detection of protein aggregates, such as p62, LC3 or TDP-43, is also more frequent in HIV-IBM. While the morphology of HIV-IBM closely mimics IBM without associated HIV infection, therapeutic responses diverge between the two disorders, as HIV-IBM patients have been observed to sometimes benefit from immunosuppressant treatment [47]. Interestingly, a similar pattern of disease is seen in patients infected with human T-lymphotropic virus-type I (HTLV-I) [54]. Although rare, HTLV-1 primarily infects T cells and is linked to the development of leukemia. In a study of 11 patients from Japan, HTLV-1 infection associated with IBM demonstrates a similar clinical phenotype and pathomorphology to HIV-IBM.

Taken together, HIV-associated myopathy displays an intricate association to IBM. A clinical progression to an IBM-like phenotype in HIV-associated myopathy argues for a shared immunopathology.

## Viral infections and IBM

IBM has not only been described in the context of HIV, although this is the most well-documented associated viral infection. IBM also occurs with other chronic viral diseases such as hepatitis C [30, 71, 72]. Again, it is unlikely to be the direct effect of the hepatitis virus resulting in the observed phenotype. Uruha et al. describe a large proportion of patients with antibodies against the hepatitis C virus (HCV) (28%) in IBM as compared to age-matched controls with IIM (4.5%) [72]. To contextualize these numbers, the prevalence is 3.4% in the general Japanese population aged 60 and older. The underlying immunological link between HCV and IBM has not yet been explored, with clinical progression and the extent of pathological features being similar between HCV-IBM and IBM [72]. The upregulation of interferon-stimulated genes (ISG), resulting in elevated levels of circulating interferons, may contribute to extrahepatic manifestations of HCV infection, such as myopathy and cognitive deficits [38, 66]. It is interesting to note that, to our knowledge, associations between IBM and the hepatitis B virus (HBV) have not been described [55]. Similar to HIV, viral chronicity in HCV results in persistent immune stimulation and exhaustion [24, 49, 56]. Consequently, the concept of immune senescence might be shared across conditions.

## Sjögren syndrome and IBM

**Fig. 3:**
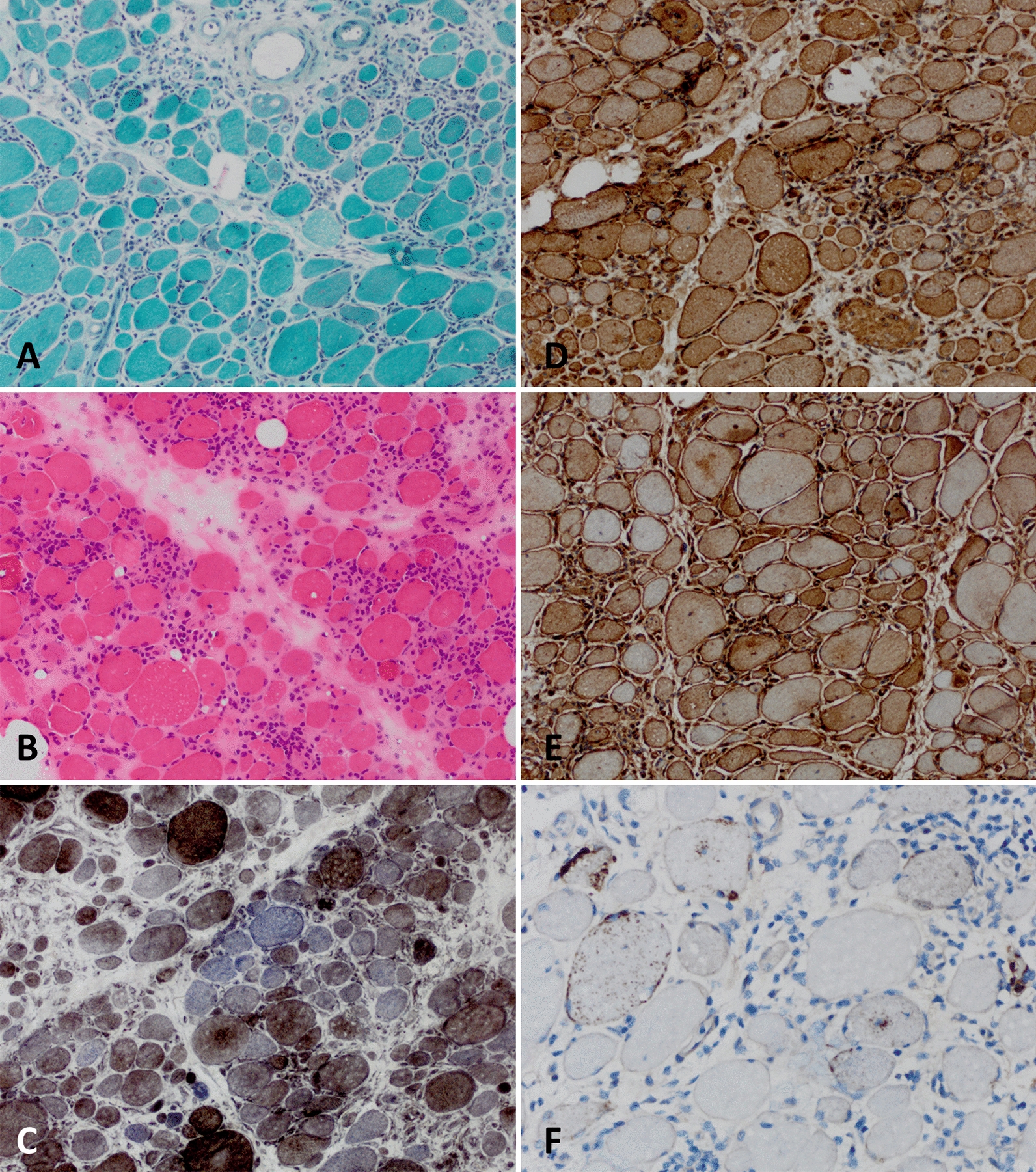
Sjögren syndrome-associated IBM. Pathomorphological characteristics of IBM associated with Sjögren syndrome as seen on muscle biopsy. (**a**) Myopathic picture with pronounced fiber size variation with hypotrophic and hypertrophic fibers and a diffurse, dense endomysial lymphocytic infiltrates. Gömöri trichrome staining (× 200). (**b**) Pronounced fiber size variation with hypotrophic and hypertrophic fibers as well as internalized nuclei, myofiber necrosis and endomysial lymphocytic infiltrates and rimmed vacuoles. H&E staining (× 200). (**c**) Presence of COX-negative, SDH-positive myofibers. COX-SDH staining (× 200). (**d**) Myofibers display strong sarcolemmal (and sarcoplasmic) positivity for MCH class I. MHC class I staining (× 200). (**e**) Myofibers display sarcolemmal (and sarcoplasmic) positivity for MHC class II. MHC class II staining (× 200). (**f**) Coarse p62^+^ autophagic material mostly localized in vacuoles and subsarcolemmaly with some fibers showing more fine granular autophagic material (× 400). *COX*  cytochrome oxidase immunohistochemistry; *H&E*  hematoxylin and eosin; *IBM*  inclusion body myositis; *MHC*  major histocompatibility complex; *SDH*  succinate dehydrogenase

Sjögren syndrome (SjS) is characterized by chronic autoimmunity directed against exocrine glands, mainly the salivary and lacrimal glands. The clinical phenotype ranges from isolated sicca syndrome to a systemic disease with musculoskeletal pain and fatigue [9]. Interestingly, associations between IBM and SjS have been reported in the past. In a study from Greece, three (0.6%) out of 518 patients with SjS were also diagnosed with IBM [37]. This data is contrasted by another study that observed only one IBM patient in a cohort of 1320 patients with SjS [14]. Interestingly, rheumatological comorbidities are frequent in IBM patients. In a study of 3160 patient with probable IBM in Norway, coexisting rheumatological disease was reported in 25% of cases with SjS accounting for 10% [19]. Moreover, anti–Sjögren-syndrome-related antigen A (SSA) autoantibodies were detected in 20% of IBM patients [19]. In a large retrospective study from the United States, SjS was reported in 6% of IBM patients rendering this group of IIM to be 6.2 times more likely to have SjS than population-based controls [55]. In contrast, lupus erythematosus, systemic scleroderma and rheumatoid arthritis were more frequent in IIM than in IBM [55]. At the pathomorphological level, IBM associated with SjS closely resembles ‘pure’ IBM with endomysial infiltration, muscle fibre size variation and COX-negative myofibers (Fig. 3).

There are three immunological links between SjS and IBM warranting investigation. First, both IBM and SjS are associated with HLA-DR3, a component gene-allele of the AH8.1 ancestral haplotype [57, 63]. HLA-DR3 is known to predispose to autoimmune disease. Indeed, in a study of 57 IBM patients from Australia, HLA-DR3 carriers had lower quadriceps strength and a more rapid decline, suggesting that the HLA haplotype influences disease progression [57]. In a smaller study investigating the association of IBM and SjS, 6 patients with co-existing diseases were carriers of the HLA-DR3 haplotype [63]. Although limited to a small number of patients, these results argue for a common genetic predisposition linking IBM and SjS.

Further, both SjS [20, 21] and IBM [26, 28] are associated with T cell large granular lymphocytic leukaemia. Indeed, a recent study demonstrated that muscle invasion by large granular lymphocytes was present in 15/15 IBM patients but only in 1 out of 28 PM or DM patients, thus establishing clonal T cell expansion as a characteristic hallmark of IBM. Interestingly, clonal expansion of T cells was also recently described in SjS, although more prominently featuring the CD4^+^ than the CD8^+^ T cell compartment [34, 36, 79]. As such, clonal expansion of cytotoxic CD4^+^ T cells correlated with glandular dysfunction in SjS patients [36]. It may therefore be suggested that the immune architecture of IBM and SjS facilitates T cell failure resulting in expansion of a cytotoxic population, providing a potential link between these disorders.

Lastly, although highly specific for IBM, anti-cN-1A-antibodies are also detected in ~ 12% of SjS (range: 7–19%) and ~ 10% of lupus erythematosus (range: 6–21%) [32, 62]. While the diagnostic usefulness of anti-cN-1A-antibodies has been well investigated, the pathogenic role of this antibody in IBM is yet to be elucidated. A recent study reported the first evidence that anti-cN-1A-antibodies influence IBM pathomorphology, as these antibodies were associated with p62 aggregation and more pronounced macrophage infiltration in an in vivo passive immunization model [69]. In contrast, it remains unknown if anti-cN-1A-antibodies contribute to the pathophysiology of SjS (or lupus erythematosus).

Taken together, the association of IBM and SjS is characterized by distinct immune features, including the HLA-DR3 haplotype, an association with T cell large granular lymphocytic leukaemia and the anti-cN-1A-antibody. The extent of these co-occurrences appears not to be shared by other rheumatological disorders and argues for a specific link between the immunopathology of IBM and SjS.

## Muscular sarcoidosis, granulomatous myositis and IBM

**Fig. 4:**
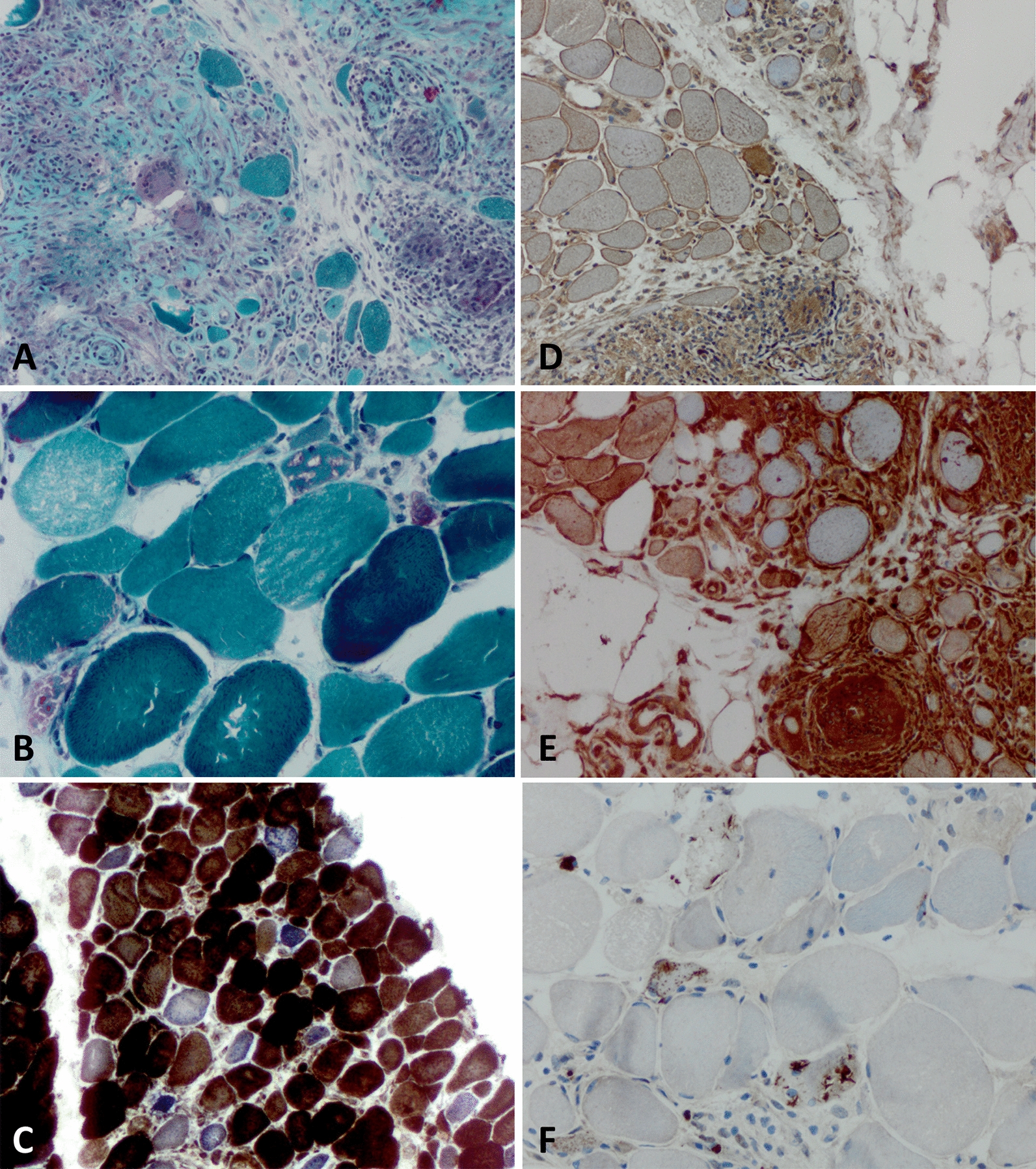
Granulomatous myositis overlapping with IBM. Pathomorphological characteristics of granulomatous myositis overlapping with IBM as seen on muscle biopsy. (**a**) Granulomatous inflammatory infiltrates with sparse giant cells and intermingled single myofibers. Gömori trichrome staining (× 200). (**b**) Severe fiber size variation with hypotrophic and hypertrophic fibers as well as multiple rimmed vacuoles. Gömori trichrome staining (× 400). (**c**) Presence of multiple COX-negative and SDH-positive myofibers. COX-SDH staining (× 200). (**d**) Myofibers display sarcolemmal (and sarcoplasmic) positivity for MHC class I. MHC class I staining (× 200). (**e**) Myofibers display sarcolemmal (and sarcoplasmic) positivity for MHC class II. MHC class II staining (× 200). (**f**) Coarse p62^+^ autophagic material mostly localized in vacuoles and subsarcolemmaly with some fibers showing more fine granular autophagic material (× 400). *COX*  cytochrome oxidase immunohistochemistry; *IBM*  inclusion body myositis; *MHC*  major histocompatibility complex; *SDH*  succinate dehydrogenase

Sarcoidosis is characterized by noncaseating granulomas in affected organs [3]. Involvement of skeletal muscle is called muscular sarcoidosis or sarcoid myopathy [3, 11]. This manifestation infrequently accompanies sarcoidosis and presents with a highly variable clinical phenotype, ranging from acute myositis (in younger patients) to a pseudomyopathic form (in older patients) [3]. The nomenclature of muscular sarcoidosis is complicated by granulomatous myositis, a disease entity characterized by granulomas in striated muscle that is most often associated with sarcoidosis, but not exclusively [61]. Here, we will use the latter term—granulomatous myositis—to describe muscle inflammation associated with the presence of noncaseating granulomas. The association between granulomatous myositis and IBM has been reported since 1986 [17] and was replicated in a panoply of studies, most recently in a cohort of 23 patients from France [18]. Regarding the muscle biopsies, there is a striking co-occurrence of typical, noncaseating granuloma formation in perimysial and, to a lesser extent, endomysial areas, with characteristic IBM features (Fig. 4) [18, 44]. Among 2952 consecutive muscle biopsies, Vattami et al. identified 27 patients with IBM and 6 with pulmonary sarcoidosis. Out of the 27 IBM patients, two had sarcoidosis and out of the 6 patients with pulmonary sarcoidosis, two had IBM [73]. The frequency of the association between granulomatous myositis and IBM was corroborated in a study from Japan describing granuloma formation in 4 out of 15 IBM patients [65]. Recently, a study group from France provided an interesting approach by comparing a cohort of patients with granulomatous myositis to a control group of IIM and a group of IBM [18]. Here, almost half of patients diagnosed with granulomatous myositis matched the diagnostic criteria of IBM [18]. These patients responded poorly to immunosuppressive treatment, similar to IBM [18]. The presence of congophilic inclusions and p62-positive deposits was associated with a treatment-refractory course of disease in granulomatous myositis [1]. Further, patients with granulomatous myositis that fulfilled diagnostic criteria for IBM had anti-cN-1A-antibodies in 43% of cases, a frequency comparable to ‘pure’ IBM patients [18]. One difference between granulomatous myositis and IBM is frequent extramuscular involvement in the former condition. All patients with granulomatous myositis demonstrated involvement of joints, lung, kidneys or skin in the previously mentioned study [18]. To date, potentially owing to the rarity of the two disorders, the immunological link between granulomatous myositis and IBM remains unclear. However, dissecting the immune overlap might be of value towards better understanding both conditions and their intricate interplay (Fig. 4).

## T cell exhaustion and immune senescence at center stage

Loss of physiological robustness is a hallmark of biological aging. Almost every organ and system in the body is affected, including the immune system. Although lacking a conclusive definition, the term immunosenescence is employed to summarize the age-dependent deterioration of the immune system [58, 80]. Immune senescence might serve as conceptual framework that explains the diminished responses to vaccines, frequent occurrence of cancer and chronic inflammatory disease, and vulnerability to infections that are observed in old age [58, 78, 80]. The specific immune phenotype of certain immune cells is also altered during aging. As such, terminally differentiated effector T cells may expand in aged individuals [41, 75, 80]. These cells are characterized by loss of CD27 (and CD28) and expression of CD57, low or absent proliferative capacities and secretion of proinflammatory cytokines such as IL-6 or TNFα [67].

Two independent datasets identified KLRG1 as a marker of highly differentiated cytotoxic T cells in skeletal muscle of IBM patients and demonstrated that these cells are absent in IIM, not including IBM [23, 27]. This cell population demonstrates a cytotoxic signature (expression of various granzymes and perforins) and a highly differentiated T cell phenotype (KLRG1^+^, CD244^+^, T-bet^+^, CD57^+^/CD28^−^, CD62L^−^) [23, 27]. KLRG1 is an inhibitory receptor of the C-type lectin-like family identified both on NK cells and T cells [68]. It is important to note that there are (at least) two different KLRG1^+^ T cell populations: (i) TEMRA cells being KLRG1^+^CD57^+^CD27^−^CD28^−^CCR7^−^CD12^dim^ [27] and (ii) central memory T cells that are KLRG1^+^CD57^−^CD27^+^CD28^+^CCR7^+^CD127^+^ [35]. The presence of terminally differentiated CD8^+^ T cells has been demonstrated both in blood and muscle of IBM patients and was replicated across studies [23, 27]. This observation is important as it might provide an suitable explanation for the treatment-refractory nature of IBM, given that these terminally differentiated CD8^+^ T cells do not readily respond to contemporary immunosuppressants [25, 27]. Consequently, therapeutic approaches directed at these cells are currently being explored [53]. One aspect that might shape therapeutic strategies is the persistence of degenerative features even after the amelioration of inflammation. In one example, immunodeficient mice were xenotransplanted with human IBM muscle and human T cells were cleared using an anti-CD3-antibody [10]. In this model, degenerative patterns as exemplified by rimmed vacuoles persisted despite normalization of MHC-1 expression after T cell depletion [10].

T cells that are exposed to chronic stress (i.e. antigen exposure) might develop a specific, exhausted phenotype [76]. In this context, PD1 is of importance. PD1 is an inhibitory receptor regulating T cell response to chronic stimulation including persistent inflammation but also cancer [40]. We and others have previously demonstrated that the PD1 signaling pathway might contribute to T cell exhaustion in IIM. T cells in IBM were PD1-positive and we observed a particular accumulation of senescent T cells in IBM muscle [40]. This effect was not unique to IBM, but also observed in IMNM and immune checkpoint inhibitor-related myositis.

However, it is intriguing to note that cellular senescence contextualized by chronic inflammation has been a recurring theme across IBM and its associated disorders. More specifically, we observed that HIV promotes immune senescence and is intimately linked to IBM. HIV is known to induce chronic immune activation and hyperstimulation of T cells leading to premature immune senescence [7], thereby potentially shifting the age of onset to a younger age for IBM patients [49]. This feature, and its association to IBM is potentially shared by HCV, another chronic viral infection discussed to induce T cell exhaustion [49, 56]. This link has also been replicated in SjS, with salivary gland progenitor cells demonstrating features of senescence, as evidenced by p16 expression [74]. These features correlated with immune cell infiltration and disease severity. We speculate that IBM and associated pathologies develop in a permissive environment that promotes early T cell exhaustion and senescence, which cumulates in the accrual of terminally differentiated cells mediating autoimmunity against skeletal muscle. T cell senescence is unable to explain the full extent of IBM pathophysiology, but it might provide a framework for the treatment-refractory course of disease and the characteristic expansion of terminally differentiated, cytotoxic CD8^+^ T cells present in blood and muscle.

## Outlook

**Fig. 5:**
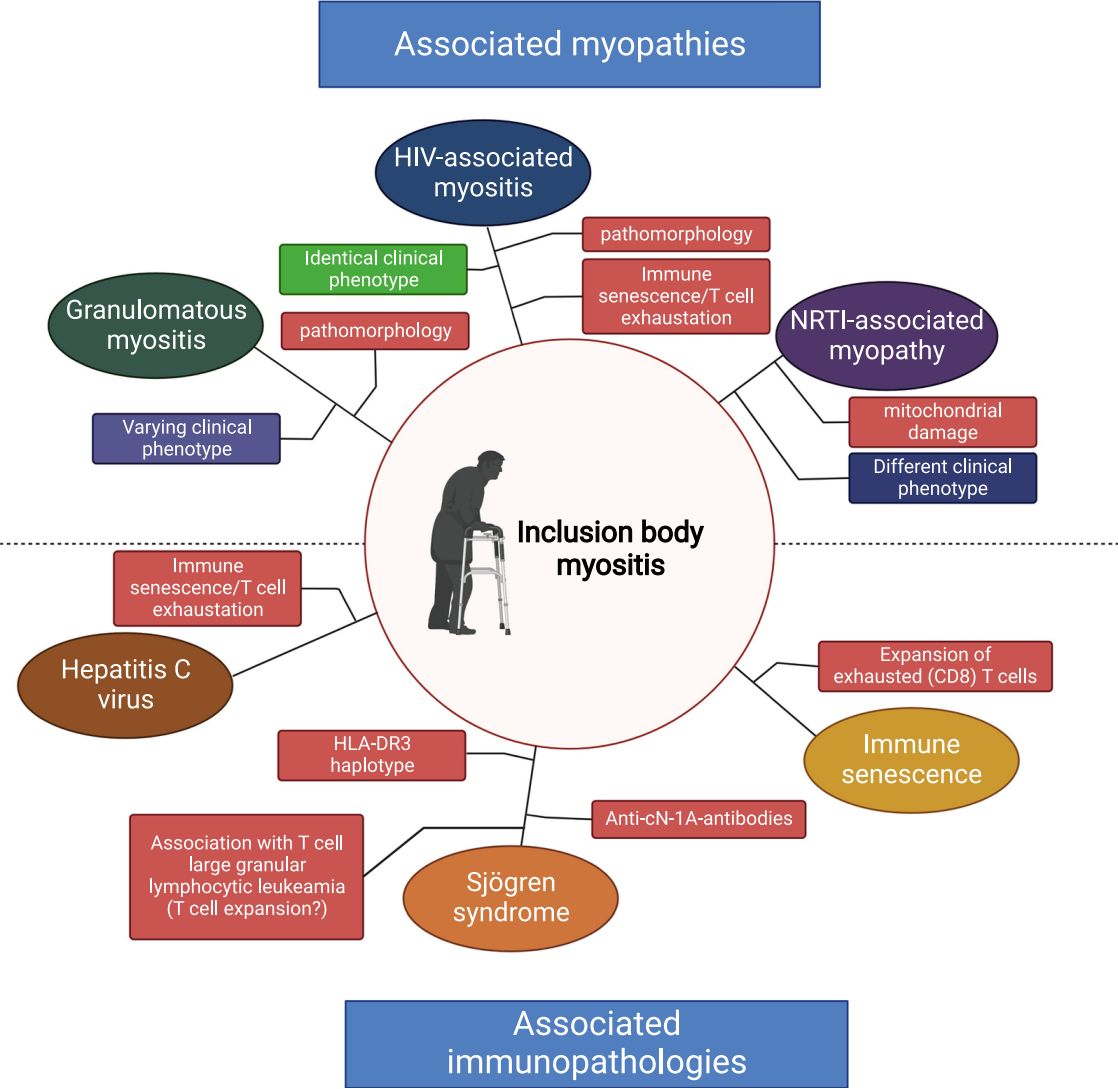
Associated inflammatory myopathies and immunopathologies in IBM. In the upper half, myopathies with associated features to IBM are displayed. Red boxes show shared disease features in the clinical phenotype. In the bottom half, immunopathologies with overlapping features are displayed

Similar histopathological patterns are seen across a range of conditions, such as SjS, HIV-associated myositis and granulomatous myositis (Fig. 5). This association is contextualized by interesting commonalities between IBM and immune senescence and the likely pathological expansion of terminally differentiated CD8^+^ T cells. Future studies aimed at understanding how IBM and associated conditions co-occur might shed light on the intricate pathophysiology of IBM. To dissect this interplay, research might focus on studying autoimmunity across IBM, HIV-IBM, SjS and granulomatous myositis to identify similarities and differences between these disorders.
